# Intravitreal Fluorinated Gas Preference and Occurrence of Rare Ischemic Postoperative Complications after Pars Plana Vitrectomy: A Survey of the American Society of Retina Specialists

**DOI:** 10.1155/2012/230596

**Published:** 2012-09-11

**Authors:** Eric J. Sigler, John C. Randolph, Steve Charles, Jorge I. Calzada

**Affiliations:** Division of Vitreoretinal Surgery, Charles Retina Institute, Memphis, TN 38119, USA

## Abstract

*Objective*. To perform a survey of the American Society of Retina Specialists (ASRS) regarding the use of vitreous cavity fluorinated gas as an adjunct to pars plana vitrectomy for retinal detachment or macular hole repair. *Methods*. A multiple-choice online questionnaire was administered to members of ASRS. Physician experience, gas preference for vitrectomy, and categorical estimate of observation of blinding postoperative ischemic events were recorded. *Results*. 282 questionnaires were completed. Mean years in vitreoretinal practice were 15 ± 10. A decrease in yearly vitrectomy volume was associated with increased number of years in practice (*P* = 0.011). Greater than 95% of respondents preferred fluorinated gas to air alone for both retinal detachment and macular hole repair. 38% of respondents reported at least one observation of a blinding ischemic postoperative event. Overall estimated incidence of blinding postoperative ischemic event was 0.06 events/year in practice. *Conclusions*. Currently, C3F8 and SF6 are the postoperative gas preference for ASRS respondents, in contrast to previous North American surveys. The occurrence of blinding ischemic events appears unrelated to number of years in practice, was reported by less than half of those surveyed, and has occurred at an infrequent rate of approximately once for every ten years of practice for those observing the phenomena.

## 1. Introduction

Retinal detachment (RD) and macular hole (MH) surgery frequently involve the use of intravitreal gas. The use of intravitreal air injection for the repair of retinal detachment (RD) was first described by Ohm in 1911 [1] and later by Rosengran [2]. 

The subsequent search for an ideal intravitreal gas with the ability to be injected in small volumes, inert, insoluble in aqueous media, nontoxic, and have a duration of presence longer than air lead to the use of sulfur hexafluoride (SF6) [3], and perfluorinated short-chain carbon compounds (C2F6, C3F8, C4F8, and C4F10) [4, 5]. These compounds draw dissolved vitreous nitrogen, oxygen, and carbon dioxide into the infused bubble at a greater rate than the passive outward diffusion of fluorinated gas, which leads to expansion of the initial gas volume. 100% C3F8 expands to four times its initial volume over 96 hours and is present for approximately 35 days [5–7]. 100% SF6 expands 2–2.5 times its initial volume over 48 hours and maintains an effective presence for 7–10 days [5, 6]. High gas surface tension and buoyancy allow reapposition of the neurosensory retina to the underlying retinal pigment epithelium and prevent influx of additional vitreous fluid into the subretinal space through retinal breaks.

The use of vitreous cavity fluorinated gases (VCFG), particularly SF6 and C3F8, have become an integral part of many scleral buckling (SB) and pars plana vitrectomy (PPV) procedures [8–11]. Previous investigations regarding physician preference regarding FG in vitreoretinal surgery have been reported showing roughly equal distributions of plain air, SF6, or C3F8 for uncomplicated RD cases and a trend toward longer-acting FG for increased detachment complexity [12, 13]. A recent Japanese survey including over 150,000 surgeries indicated a preference for SF6 in both RD and MH cases, with intraocular pressure (IOP) rise being a common complication (15%). We have observed the occurrence of infrequent acute (within 72 hours) postoperative intraocular pressure elevation and at least two cases of ischemic optic neuropathy or central retinal artery occlusion resulting in no light perception following PPV with adjunctive expansile gas. No recent North American study, to our knowledge, addresses current trends in type of FG, concentration preference, or occurrence of these infrequent VCFG-related complications. The purpose of the present study was to survey vitreoretinal specialists regarding their preference for VCFG as an adjunct to vitreoretinal surgery (PPV or SB) and their observation of complications potentially associated with the use of expansile gases, such as IOP elevation and vascular occlusions or other blinding ischemic events (IE).

## 2. Methods

**Figure 1:**
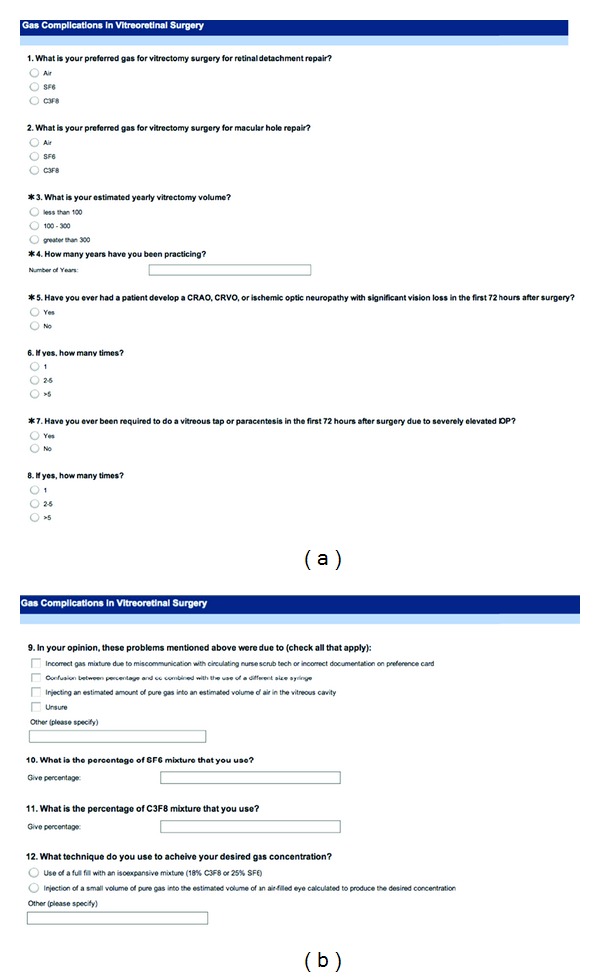
Online survey completed by American Society of Retina Specialist Members. 12-item questionnaire regarding postoperative intravitreal gas preference, vitreoretinal experience, and observation of rare complications.

The study conformed to the tenets set forth in the Declaration of Helsinki and was exempt from IRB approval. A 12-item questionnaire (Figure 1) was designed to assess physician preference for VCFG related to retinal reattachment procedure, MH repair, number of years in practice, and history of observation of complications. A list of e-mail addresses of members of the American Society of Retina Specialists (ASRS) was obtained from ASRS e-mail directory. 846 vitreoretinal specialists were available from the directory. A free online survey (Figure 1) was constructed using Survey Monkey (Palo Alto, CA, USA). Members were petitioned via email for participation in the survey. Results were tabulated over a one-month study period from 7/2011–9/2011. IE was defined either as central retinal artery occlusion, central retinal vein occlusion, or ischemic optic neuropathy resulting in dramatic permanent decrease in vision occurring within 72 hours of a PPV with VCFG. No physician identifying information was recorded in the survey or required to participate in the web-based survey. Power calculation (alpha = 0.05, sigma = 2) was performed for all planned statistics prior to study initiation and indicated a minimum sample size of 158 responses. Results were tabulated and data analyzed using JMP 9 (SAS, Cary, NC, USA). Statistical significance was considered present for *P* < 0.05. 

## 3. Results

282 participants completed the survey (33.3% response rate). A summary of respondent surgical volume and years in practice is demonstrated in Table 1. Increased number of years in practice was inversely correlated to vitrectomy volume via logistic regression (*P* = 0.0113). There were no significant correlations between VCFG choice for RD repair versus MH and number of years in practice. VCFG preference by procedure and gas concentration is demonstrated in Table 2. A significantly larger proportion of respondents reported the use of SF6 for MH than for RD (*P* < 0.001). There were no reports of the use of gases other than air, C3F8, or SF6. Analysis of the subgroup of respondents using air revealed no significant difference in years of practice experience (*P* = 0.431). Regarding intraoperative technique of air-gas exchange, 90% (*n* = 239) of respondents used a “full fill” with an isoexpansile mixture, 9% (*n* = 25) use injection of pure VCFG into an estimated eye volume to achieve a desired concentration, and 1% (*n* = 3) manually dilute pure gas in a large syringe. 

A summary of IOP-related complication and vitreoretinal specialist experience is demonstrated in Table 3. Logistic regression revealed no significant correlation between report of at least one IE and number of years in practice (*P* = 0.089), but a significant positive correlation between number of years in practice and at least one postoperative therapeutic paracentesis (*P* < 0.001). 4200 cumulative years of vitreoretinal practice were reported. By calculating the number of responses within each category as follows: one = 1, 2–5 = 5, >5 = 7 we estimated the total reported cases of IE as 265. The estimated observed rate of IE per year of vitreoretinal practice, therefore, was 0.06. Only 38% of respondents, however, reported such events. Among respondents reporting at least one IE, the estimated yearly rate was 0.15, and there were no significant difference in mean years in practice among respondent groups. No correlation was found between technique of gas preparation and either performance of therapeutic paracentesis (*P* = 0.622) or observation of IE (*P* = 0.318). 36% (*n* = 60) reported the perceived cause of postoperative IOP-related complications as communication error with personnel preparing VCFG concentration, 8% (*n* = 13) reported confusion regarding resultant concentration following syringe dilution, 8% (*n* = 13) reported under estimation of the vitreous cavity volume when injecting pure gas, and 48% (*n* = 82) reported “other/unsure.”

## 4. Discussion

The results of the present study indicate that postoperative intraocular gases currently in use by ASRS members are predominantly C3F8 and SF6 in isoexpansile mixtures. Air was infrequently used although unrelated to years of practice experience. While years of practice experience was not related to choice of SF6 or C3F8, SF6 was the preferred VCFG for PPV in MH repair. This likely reflects the theoretically less required VCFG duration for MH compared to RD. For RD repair, an equal proportion of ASRS members use SF6 and C3F8. These findings are consistent with a large recent survey of Japanese vitreoretinal specialists, [14] but differ from previous results [12, 13] that indicated relatively equal distributions of air, C3F8, and SF6 after PPV for uncomplicated RD and MH. This likely reflects the trend in availability, long term knowledge of outcomes, physician training, and additional experience with postoperative VCFG over the past two decades. The vast majority of ASRS members reported using a “full fill” of “isoexpansile” gas (18% C3F8 or 25% SF6), which is also the preference of the authors. The majority of remaining respondents reported injecting a known volume of pure gas into an estimated volume of vitreous cavity air. While this method may lead to a larger variation in actual induced postoperative concentration, and theoretically lead to more postoperative IOP-related events, we did not observe this relationship. In fact, the precise resultant concentration of immediate postoperative VCFG may be impossible to determine, and commonly used “isoexpansile” concentrations are actually prone to slight expansion (18% C3F8 and 20–25% SF6) as true isovolumic gas-air fractions are actually closer to 12% and 18%, respectively [15, 16]. Considering that resultant postoperative concentrations rarely exceed 95% of air-gas exchange flush volumes, [17, 18] true isovolumic gas fractions may actually be approximated *in vivo*. 

Although not a primary goal of the study, our results indicate that among the surveyed ASRS members, the majority of vitreoretinal specialists perform between 100 and 300 PPVs each year. Interestingly, respondents with more years of vitreoretinal practice had lower average yearly volume. This may indicate a higher proportion of non-PPV procedures (such as SB) in older vitreoretinal specialists, the performance of less surgery with age, or increased office time or focus on medical retinal disease due to decreasing reimbursement trends in the United States. Many confounding variables, particularly voluntary response bias may have impacted this statistical observation. We did not, however, find any association between surgical volume or experience on VCFG choice, technique, or incidence of complications.

As expected, dramatic postoperative IOP elevation, although rare, appears to be a phenomenon observed by most vitreoretinal surgeons. Nevertheless, up to 40% of respondents failed to report any observation of the need for therapeutic paracentesis after PPV with VCFG. We could not, however, find any statistical association between technique or experience with the occasional need for paracentesis. This observation may be due in part to a potentially wide variety between methods for treating postoperative IOP elevation, variation in individual IOP threshold for performing a paracentesis, different timing and method for IOP measurement after surgery, perioperative use of antiglaucoma medications, and several other potential confounding variables. Previous reports have indicated mixed results regarding postoperative IOP elevation within the first 24 hours following vitreoretinal surgery, [19, 20] but medical management is required in a relatively small, 4–30% [19–22] of patients. SB with VCFG or silicone oil, diabetic RD undergoing SB with silicone oil, expansion of VCFG, and increased duration of retinal pathology have been implicated as risk factors for postoperative IOP spike [19–22]. A 1989 prospective study [23] of over 200 vitrectomy cases identified concomitant SB, gas expansion, trabecular meshwork obstruction, and simultaneous lensectomy as potential mechanisms of IOP elevation after PPV, and reported 11% requiring surgery. 

Although IE due to expansion of postoperative gas has been rarely reported, [24–26] predominantly in association with postoperative atmospheric pressure decline and anesthetic gases, [24, 25, 27] it is difficult to determine its true incidence. Based on the current study we estimate that this observation may occur between one and two times for each 20 years of vitreoretinal practice. Again, many confounding variables including use of retrobulbar anesthesia, voluntary response bias, variability in postoperative care, and nuances of intraoperative air-gas exchange technique make this estimate difficult to assess adequately. The largest contingent of vitreoretinal surgeons reporting at least one IE reported being unsure of the underlying cause. A large proportion (38%) observing IE reported a communication error with a circulating staff member as the cause of VCFG overfill. We therefore stress the importance of close staff observation and advocate physician mixing and verification of gas concentration prior to vitreous cavity filling. We hypothesize that IE results from mechanical compression of the central retinal artery and vein during sustained IOP elevation after a full vitreous cavity fill with expansile gas due to inaccurate percentage calculations, or inaccurate volume estimates of eyes with relatively small vitreous cavity volumes. 

We conclude that there appears to be a current preferential use of VCFG compared to plain air for RD and MH repair. While techniques for gas preparation differ, two predominant methods (1) using a “full fill” with “isoexpansile gas” or (2) injection of a known volume of pure gas into the estimated vitreous cavity volume) comprise the majority of currently used methods and do not differ with respect to potential dramatic postoperative IOP elevation or IE.

## Figures and Tables

**Table 1:** American Society of Retina Specialists survey respondent characteristics.

Estimated average yearly vitrectomy volume {Category—*n* (%)}	Number of years in practice
<100—45 (16)	19 ± 11*
100–300—202 (72)	15 ± 10
>300—35 (12)	13 ± 8*
Overall—282 (100)	15 ± 10

*Values significantly different from overall mean (*P* = 0.013) via one-way ANOVA, overall values represent 33% ASRS response rate.

**Table 2:** ASRS member preferred intraocular gas type as adjunct to pars plana vitrectomy procedure*.

Procedure	Air *n* (%)	SF6 *n* (%)	C3F8 *n* (%)
Retinal detachment	14 (5)	134 (48)	134 (48)
Macular hole	6 (2)	160 (57)^†^	116 (41)
Gas concentration	All 100%	22 ± 9.7 (*n* = 252)	15 ± 9.4 (*n* = 262)

*Responses to online survey, *n* = 282, ASRS = American Society of Retina Specialists, for gas concentration, mean ± standard deviation of concentrations reported as continuous variable among *n* positive respondents for each gas category.

^†^Significantly greater than for retinal detachment via chi^2^ (*P* < 0.001).

**Table 3:** American Society of Retina Specialists survey results for intraocular pressure-related events after pars plana vitrectomy with postoperative fluorinated gas.

Event	Yes *n* (%)	No *n* (%)	If yes, how many times? (category = *n*)	Mean years in Practice* (mean ± SD)
Blinding ischemic event (CRAO, ION)	107 (38)	175 (77)	1 = 58	14 ± 4.7*
			2–5 = 40	19 ± 5.3*
			>5 = 9	19 ± 9.9*
Overall years in practice* (mean ± SD)	17 ± 5.6	14 ± 5.1	17 ± 5.6	15 ± 10

Therapeutic paracentesis	217 (62)	65 (23)	1 = 51	12 ± 11^†^
			2–5 = 90	15 ± 10
			>5 = 76	22 ± 8.8^†^
Overall mean years in practice (mean ± SD)	16 ± 3.8^†^	12 ± 4.8	17 ± 5.6	15 ± 10

*No significant differences between means via two sample *t*-test for yes versus no (*P* = 0.089) or via one way ANOVA for “yes” subgroup (*P* = 0.161), SD = standard deviation.

^†^Significantly different from “no” via two sample *t*-test for “yes” versus “no” (*P* = 0.001), and from overall mean via one-way ANOVA for “yes” subgroup.
